# Compensation for Adolescents’ School Mental Load by Physical Activity on Weekend Days

**DOI:** 10.3390/ijerph13030308

**Published:** 2016-03-09

**Authors:** Michal Kudláček, Karel Frömel, Lukáš Jakubec, Dorota Groffik

**Affiliations:** 1 Faculty of Physical Culture, Institute of Active Lifestyle, Palacký University Olomouc, Olomouc 77111, Czech Republic; karel.fromel@upol.cz (K.F.); lukas.jakubec@upol.cz (L.J.); 2 Department of Leisure Studies, Faculty of Physical Culture, Palacký University Olomouc, Olomouc 77111, Czech Republic; 3 The Jerzy Kukuczka Academy of Physical Education, Katowice 40-065, Poland; d.groffik@awf.katowice.pl

**Keywords:** mental health, heart rate, monitoring, accelerometer, health education, physical literacy

## Abstract

*Introduction and objective:* Increasing mental load and inadequate stress management significantly affect the efficiency, success and safety of the educational/working process in adolescents. The objective of this study is to determine the extent that adolescents compensate for their school mental load by physical activity (PA) on weekend days and, thus, to contribute to the objective measurement of mental load in natural working conditions. *Methods*: A cross-sectional study was conducted between September 2013 and April 2014. A set of different methods was employed—self-administered questionnaire (IPAQ-long questionnaire), objective measurements—pedometers, and accelerometers (ActiTrainers). They was distributed to 548 students from 17 high schools. Participants’ mental load was assessed based on the difference between PA intensity and/or physical inactivity and heart rate range. *Results*: The participants with the highest mental load during school lessons do not compensate for this load by PA on weekend days. *Conclusions*: Adolescents need to be encouraged to be aware of their subjective mental load and to intentionally compensate for this load by PA on weekend days. It is necessary to support the process of adopting habits by sufficient physical literacy of students, as well as teachers, and by changes in the school program.

## 1. Introduction

The accelerating technological development and changes in educational systems across the world place increased educational demands on the younger generation [1,2,3]. Positive effects of such changes, however, give rise to negative impacts on the lifestyle of youth [4]. Increasing mental load and inadequate stress management, in conjunction with sedentary behavior, are the most serious problems in health promotion in younger generations. These issues are also closely associated with the efficiency and success of the educational/working process, as well as with security and adoption of necessary working habits for a future profession. We have sufficient evidence that youth engage in less physical activity (PA) on weekends than on school days [5,6,7,8]. Nonetheless, we do not know how adolescents react to their previous school mental load during weekend days.

It is well established that physical activity (PA) is an effective prevention against non-communicable diseases and, consequently, improves health [9]. In addition to the physical and physiological benefits of PA, there are numerous psychological benefits that have been identified, with the most evidence on depression and anxiety [10,11,12,13,14]. Depression is a common mental health problem in adolescents worldwide [10], with an estimated one-year prevalence of 4%–5% in mid- to late adolescence [15,16]. PA can indirectly improve subjective well-being and life quality by preventing disease and premature death, and there has recently been an increasing interest in its direct role in the prevention and treatment of mental health problems. We mentioned the most common health problems—depression and anxiety—but there are additional issues that benefit as well. The list of mental health issues should also include stress reactivity, subjective well-being, emotion and mood, self-esteem and self-perception, sleep quality, and cognitive performance [17,18,19].

A decreasing trend in the level of physical activity throughout the population is due to the sedentary behavior of youth and adults, which is a rapidly developing area of research. Most of the evidence of young people has focused on screen time and TV viewing [20,21]. It seems to be a vicious circle because individuals with poorer mental health choose to be more sedentary [22,23,24].

Sedentary behavior leads to poorer mental health, and there is a visible gap in the evidence about the sedentary behavior in relation to mental load at school.

Mental load (not only at school) is related to mental fatigue and can be classified as the amount of mental work using cognitive processes. Excessive mental load can lead to mental fatigue, which represents a failure to complete mental tasks that require self-motivation and internal cues in the absence of demonstrable cognitive failure or motor weakness [25]. There is a specific need to compensate for this excessive mental load by PA within school-time or after school, as well as within work-time or after work.

School can be a stressful environment if the educational process is not managed in a physically-active and physically-friendly way. There is considerable cross-sectional evidence suggesting that PA of moderate-to-vigorous intensity represents a natural, inexpensive, and effective means of coping with mental stress and excessive mental load. Negative aspects of excessive mental load, and the interactions of physical and mental activity were well described in the study by Singh *et al.* [26]. Their results indicate that the individual’s physical capacity decreased with mental load. Regular PA is thought to be associated with stress reduction and better mood, which may partly mediate associations between depression, stress, and other health outcomes [27,28,29,30]. If stress is not regularly balanced, it can lead to mental disorders and physical injuries.

According to the Center for Disease Control and Prevention [31], among the risk factors leading to a higher likelihood of mental disorders are high emotional distress, antisocial beliefs and attitudes, drug abuse, alcohol or tobacco consumption, attention deficits, hyperactivity or learning disorders, social rejection by peers, poor academic performance, and other factors. Moreover, positive influence of moderate-to-vigorous PA on school attainment is consistent throughout the literature [32,33] and can serve as an impulse for school-based PA strategies and promotion activities.

The optimal dose of PA needed to improve or sustain mental health is unknown [34]. A dose-response relationship between PA levels and physical health outcomes is well-accepted, although the exact shape of the dose-response curve is not well understood [9,35]. Physical load and mental load are expressed by increased energy expenditure; therefore, we can consider them as appropriate indicators of load intensity and volume [36].

Schools are responsible for students’ development not only in cognitive functioning but in all dimensions of their lives—physical, mental, social, and spiritual. Implementing health promotion activities and incentives that are built around individual, environmental, cultural, and organizational values is far more effective than focusing on any single dimension. These strategies might be general or more specific, such as taking an ergonomic approach or emphasizing exercise and education.

Despite the knowledge of the benefits of PA, trends in lifestyle among youth and adolescents lead to a sedentary society/population and to preferences of passive leisure time activities, leading to poorer mental health with consecutive health problems. Research on this topic is needed.

The objective of this study is to determine if adolescents compensate school mental load by physical activity on weekend days. Moreover, we aimed to contribute to the objective measurement of mental load in natural school/working conditions.

## 2. Material and Methods

### 2.1. Research Sample and Study Design

The research was conducted between September 2013 and April 2014 at 17 secondary schools in the Czech Republic (nine high, six technical, and two vocational schools) that agreed to participate in organizationally-demanding research and that cooperate with the authors’ institute. In total, 548 participants were included in the survey. In the classes, over 95% of students and their parents agreed to participate in the survey. The participants, who met the conditions of the demanding all-day monitoring using accelerometers, were included in the final sample, which contained 149 girls (age (years) 16.50 ± 1.10; weight (kilograms) 59.10 ± 9.94; height (centimeters) 166.82 ± 5.81; Body Mass Index (BMI); 21.23 ± 3.32; HRrest 64.87 ± 6.66) and 78 boys (age (years) 16.71 ± 1.18; weight (kilograms) 71.54 ± 11.84; height (centimeters) 179.06 ± 7.63; BMI 22.33 ± 3.56; HRrest 60.44 ± 6.56).

Only those participants who wore an accelerometer for at least 15 min before school, 180 min at school (excluding physical education lessons), 120 min after school, and at least 600 min and at most 1080 min, aggregately, during the entire day were included in the study. Similarly, on weekends, the participants had to wear the devices for at least 600 min and at most 1080 min per day. Due to these requirements, 321 participants were excluded from the study.

### 2.2. Measurements

The ActiTrainer^TM^ accelerometer (Florida, FL, USA) was used for PA monitoring. It measures both the PA (counts) and heart rate (HR) (beats per minute) at the same time. Furthermore, the Digi-Walker SW-700 (Yamax Co., Yasama Corp., Tokyo, Japan) pedometers and International Physical Activity Questionnaire—long form (IPAQ) were employed in the survey. Despite many limitations of energy expenditure estimation in real conditions, the combination of the heart rate monitor and accelerometer for recording PA is recommended—mainly due to their character, availability, and accessibility [37]. Moreover, synchronized employment of a heart rate monitor and accelerometer increases the accuracy of energy expenditure estimation [38,39].

There is a range of objective methods, in the area of measuring mental/cognitive load, which are strictly focused on this type of load—e.g., magnetic resonance imaginary (MRI), positron emission tomography (PET), and performance operating characteristics (POC). The other method—a relatively frequently used method—is the measurement of heart rate variability (HRV) or ECG recording [40,41,42]. However, the application of these methods in natural/real-life conditions is complicated, especially within all-day monitoring which was the main purpose of our research design. We tried to contribute to the mental load measurement verification through the combination of employed research methods and add new perspective of the mental load phenomenon and its compensation in natural conditions.

The same research team introduced the course of the survey at all of the participating schools prior to the beginning of the survey. The participants were instructed on how to monitor PA, wear and remove devices, monitor HR, and complete the recording sheets (course of the day, recording the time of particular day segments). The wearing time was set for four days from Thursday to Sunday to increase the chance of recording at least one school day and one weekend day. In addition to accelerometers, the participants wore pedometers. However, the pedometers were used for PA monitoring for the entire week. Data on participants' age, height, and weight were taken from the current school documentation (updated information about height and weight; in case of missing data, participant was measured on site). The participants were told how to measure their resting heart rate immediately after waking up in the morning (repeatedly three times). As a part of the introductory information, the participants completed the IPAQ questionnaire via the Indares Internet system (www.indares.com) [43]. After the introductory session, they began wearing the pedometers, but the PA monitoring was initiated on the following morning.

**Figure 1:**
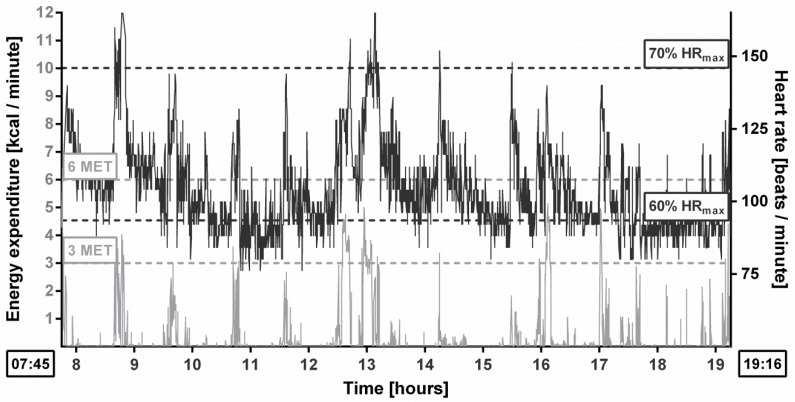
Example of individual feedback.

Participants received individual results of daily PA monitoring (time records of PA and inactivity, load in metabolic equivalents (METs) and HR ranges, energy expenditure, HR records. and step count) within two weeks from the completion of research (Figure 1).

### 2.3. Data Processing 

The participants entered the time defining periods into their recording sheets: before school, at school in accordance with the schedule of lessons and recesses, after school, and aggregately for the entire day. On weekend days, they only recorded the times of mounting and removing the devices.

To process the data (epoch length of 15 s), we used the specially developed software IntPA13. The program processes data on the duration of PA and inactivity in minutes. The level of PA intensity was determined according to a heart rate of 30%–100% maximum heart rate (HR_max_) in 10% increments and in METs in one-MET increments. To identify the heart rate ranges, we applied a universal formula to calculate maximal heart rate (for boys, HR_max_ = 220-age and for girls, HR_max_ = 226-age). The intensity levels were split into low (50%–59.9% HR_max_ < 3 METs) and moderate to vigorous (≥60% HR_max_ ≥ 3 METs). Resting metabolic rate was determined according to the formula ((473 × weight in lb.) + (971 × height in in.) − (513 × age) + 4687)/100,000 for male subjects and ((331 × weight in lb.) + (352 × height in in.) − (353 × age) + 49,854)/100,000 for female subjects. These formulas were also applied in the TriTrac R3D, the former type of accelerometer. For the transfer of count values to kcals/min and subsequently to the MET values, we used the formula (kcals/min = 0.0000191 × counts/minute × body mass in kg). The physical inactivity cut-points were found to be <25 counts per 15 s.

The strongest correlation was found between time spent in the zone ≥60% HR_max_ and ≥6 METs (r_s_ = 0.284), respectively, and time spent in the zone ≥60% HR_max_ and ≥3 METs (r_s_ = 0.288) within our all-day PA and HR field monitoring. Due to our effort of emphasizing the differences between measured HR and PA intensity, we chose as more probable indicator of mental load the level ≥60% HR_max_ and ≥6 METs.

The participants were split into four groups depending on whether they reached or exceeded the physical load at the level of 6 METs, compared with the load of 60% HR_max_, in the course of school lessons (excluding recesses and eventual physical education lessons). Characteristics of the groups:

1. Very high mental load (VHML)—PA < 6 METs a HR ≥ 60% HR_max_
2. High mental load (HML)—PA ≥ 6 METs a HR ≥ 60% HR_max_
3. Low mental load (LML)—PA < 6 METs a HR < 60% HR_max_
4. Very low mental load (VLML)—PA ≥ 6 METs a HR < 60% HR_max_

The most specific group was formed by the participants who reached the physical load of only < 6 METs within school lessons, including physical inactivity, but also concurrently, the HR load of ≥60% HR_max_. VHML individuals were the most mentally loaded students during school lessons, whereas the level of mental load in the remaining groups was difficult to objectively determine.

### 2.4. Data Analysis

Descriptive analyses were conducted to summarize the mean, standard deviations of the variables, medians, and interquartile range.

To address the main objective of the study, repeated ANOVA with the Scheffé *post hoc* test, Kruskal–Wallis test, crosstables, and effect size were established to explore the associations and differences between constructed split groups [44].

All analyses were performed using SPSS 22. Statistical significance was defined as *p* < 0.05.

### 2.5. Ethical Principles

The present study was approved by the Ethics Committee of Faculty of Physical Culture, Palacký University Olomouc under reference number 24/2012. All participants volunteered to participate and signed an informed consent form prior to data collection. All data in this study was collected anonymously.

## 3. Results

### 3.1. The Initial Level of Physical Activity (IPAQ)

Regarding the overall level of PA (MET-min/week) in the week prior to the start of the research, we found no significant differences among groups with various levels of mental load (Table 1). Additionally, the difference in sedentary time between school and weekend days was not significant.

### 3.2. Compensation for School Mental Load on Weekend Days in Terms of PA Volume (Pedometer)

**Figure 2:**
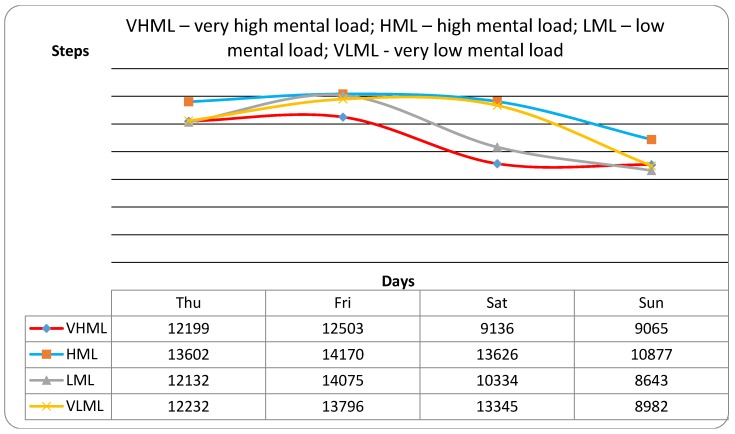
PA volume (steps/day) in participants with various levels of mental load on two school days and two weekend days.

The participants with very high school mental load do not compensate for this load by physical activity (steps/day) on weekend days, which is similar to other participants with lower mental load (F_(3227)_ = 36.03; *p* ≈ 0.000; ω^2^ = 0.161) (Figure 2). In summary, the participants reached 10,619 ± 5726 steps/day on Saturdays and 9137 ± 4695 steps/day on Sundays. On Fridays, it was 13,742 ± 5630 steps/day and 12,389 ± 4505 steps/day on Thursdays. No significant differences were found in the interaction between days and participant groups (F_(3227)_ = 1.31; *p* ≈ 0.229; ω^2^ = 0.002). The values of obtained effect sizes fortified the results of statistical non-significance.

**Figure 3:**
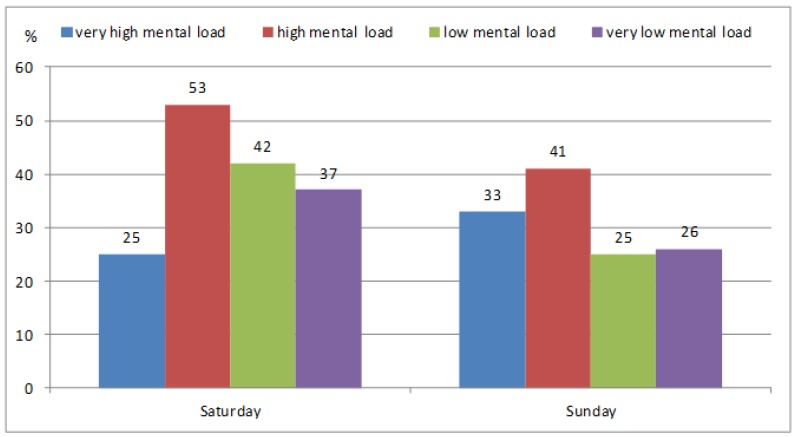
Meeting the recommendation of 11,000 steps/day on weekend days in participants with various levels of school mental load.

The recommendation of 11,000 steps/day also shows (Figure 3) that participants with very high mental load do not compensate for it by the volume of PA. In particular, the fact that only 25% of these participants meet the recommendations is alarming.

### 3.3. Compensation for School Mental Load on Weekend Days in Terms of PA Intensity (ActiTrainer)

The results based on the objective PA monitoring using the ActiTrainer accelerometer correspond to the findings obtained through pedometers. Regarding volume or intensity of PA on a monitored weekend day, we did not find significant differences between the participants of particular groups (Table 2). Only the differences in the step count per hour (steps·h^−1^) and in minutes of load of ≥3 METs (min·h^−1^) between participants with VHML and the participants with HML were significant in which participants with VHML had the least amount of PA. The values of obtained effect sizes within these categories and groups fortified the results of statistical significance. Considerable caution is warranted in interpreting the statistically significant findings, and they should not be interpreted to mean something practically meaningful [45].

The differences in time of physical inactivity on a weekend day were insignificant among the groups.

## 4. Discussion

This study provides new insight into the individual and environmental correlates of moderate-to-vigorous physical activity (MVPA) in adolescents with a specific focus on school mental load. The results of physical activity monitoring in the context of school mental load proved a lack of compensation for school mental load by physical activity. It is congruent with previous study of Svozil *et al.* [46]. The insufficiency was found in both the volume and intensity parameters. This finding is based on the volume of PA represented by the average number of steps per hour, as well as on the amount of time spent in MVPA.

A crucial part of this study was its design. The complex PA monitoring was employed, which was a “contraindication” of this study. The combination of the ActiTrainer accelerometer, the Digi-Walker SW-700 Yamax pedometer, and the International Physical Activity Questionnaire (IPAQ) resulted in strict exclusive criteria of this study. The usage of these methods was crucial because of the comparison of objectively-measured PA and subjectively-responded questionnaire, therefore we could make our study more reliable. The other reason was the analysis of PA structure. Due to the lack of literature on the issues of school education and physical activity in regard to mental load, we were forced to establish our own criteria. These criteria were based on the combination of the PA level (expressed in METs) and heart rate (expressed as % of maximum heart rate). Of the relatively large sample (*n* = 548) after applying exclusive criteria, 227 participants were included in the final sample. The lack of literature and previous research on a similar topic pinpoints the missing link between physical and cognitive elements of education.

In the area of mental load, most studies address a cognitive element, called cognitive load, and education, but without subsequent consequences and associations with compensation [47,48,49,50].

The key question of our study was the compensation for school mental load by physical activity after school or on the weekend. Fairclough *et al.* [51] also observed PA in school and outside of school. They divided a research sample into two groups—high and low activity—and then they tested these groups in various school day segments. In agreement with our findings, Fairclough *et al.* [51] stated that the greatest differences between the high and low activity groups occurred in out-of-school time. A possible explanation is the greater discretionary time available for PA and other recreational opportunities during non-school hours, which is consistent with other studies using objective PA measures [52,53,54,55].

To the best of our knowledge, this investigation is the first study to examine the associations between school mental load and the compensating effect of PA. Based on our findings, we can state that highly- and very highly-mentally loaded individuals do not compensate for this school load by an adequate amount of PA on the weekends.

Ridgers *et al.* [56] tested the concept of the “compensation hypothesis” by examining associations between time spent in various PA intensities and/or sedentary time on any given day and time spent in these activities on the following day. In contrast with our results, they stated that children appear to compensate their PA or sedentary time between days. However, the study of Ridgers *et al.* [56] was adjusted for school environment more than for comparison of weekdays and weekends. This hypothesis assumes that individuals have a biological PA regulator or “activity-stat”, such that the body employs a range of biological responses to changes in PA to maintain constant daily energy expenditure [57]. However, the study by Long *et al.* [58] found that higher school-day MVPA was associated with higher overall MVPA, without evidence that youth compensated for school-day MVPA by reducing activity outside the school day. This biological PA regulation expressed in the “compensation hypothesis” is an interesting issue, but the other research initiatives should lead towards active compensation for school mental load after school and during weekends.

The discrepancy between weekday and weekend PA is consistent with other recent work in the United States and the UK [8,59]. These studies suggested that the lower weekend activity levels may be influenced by less frequent bouts of light and more intense PA, which are possibly mediated by the greater choice of recreational (and often sedentary/passive) pursuits available to youth on weekends.

In agreement with recent studies based on meeting physical activity recommendations, adolescents do not sufficiently meet PA recommendations during weekends. Fairclough, *et al.* [60] highlighted the inadequate level of moderate PA and vigorous PA in children. The study by Verloigne *et al.* [61] confirms the same situation in the area of meeting PA recommendations across various European countries. Physical activity is substituted by sitting and inactive recreation/passive recreation and leisure time.

According to a strictly mathematical conversion by Marshall *et al.* [62] would be equivalent to 8000 steps of 60 min of MVPA. However, recommendations for adolescents by Tudor-Locke *et al.* [63] were not met. Tudor-Locke *et al.* [63] presents recommendations for PA based on the number of steps/day in relation to MVPA for 11,000 steps for girls and 13,000 steps for boys, at least five days/week.

The decline in adolescent PA is mostly consistent across various environmental settings, attributable to falls in light-intensity/habitual activity and influenced by puberty, suggesting that the inactivity of adolescents may, in part, be under biological control [64]. Ortega *et al.* [65] state that a decline in MVPA (overall change = 30 min/day) and an increase in sedentary time (overall change = 2:45 h/day) observed in their study from childhood to adolescence are of concern and might increase the risk of developing obesity and other chronic diseases later in life. This trend in physical inactivity was confirmed by Hallal *et al.* [66] by providing intercontinental comparison of various WHO regions. This transferability of physical inactivity from childhood and adolescence into adulthood is well-described in the literature [67]. The possible consequences are also visible in relation to the transfer from school to work. Accordingly, children are not stimulated towards becoming physically literate individuals. They will not be able to objectively assess their personal physical needs, and there will be no tendency to compensate for sedentary habits within their lives, *i.e.*, also within their occupation.

The current study has several limitations. First, there is no previous agreement for mental load measurement within natural working or educational settings. Future longitudinal research is needed to establish the associations and causalities between the variables. Second, the implementation of presented research design is really demanding and complicated; therefore, it should be simplified. Third, due to complex and difficult HR monitoring and recording time data, there was no documentation of subjective reasons for increased HR in individual lessons. However, the estimation of energy expenditure through accelerometers and HR monitors considerably fluctuates according to conditions and various circumstances during measurement [68]. Further research with this particular focus should be created and possible adaptations and supplementary methods should be found.

Future studies should focus on optimization of research methods with potential overlap into the all-day monitoring. There is a research gap including concurrent employment of heart rate variability measurement, subjective methods (questionnaire, interview, and/or scale), magnetic resonance imagery, positron emission tomography, performance operating characteristics, multitasking experiments, and other methods. It seems to be a real challenge to find appropriate, useful, valid, and reliable combinations of methods to investigate mental load in a daily perspective together with PA characteristics, and psychophysiological variables, including fitness level, genetic disposition, age, and environment.

## 5. Conclusions

It was confirmed that the PA on weekend days does not sufficiently compensate for the adolescents’ mental load regarding neither PA volume nor PA intensity indicators. Students at school need to be encouraged to be aware of their subjective mental load and to intentionally compensate for this load by PA on weekend days. The findings on compensation for mental load by PA should also be an essential part of the physical literacy of students, teachers, and parents. Use of the ActiTrainer accelerometers was an appropriate method of objectification of mental load in adolescents.

Measurement of mental load in natural school conditions appears to be applicable in the work process and particularly in sedentary jobs or numerous mentally-demanding management professions, such as executives, managers, and similar professional roles. Interpreting accelerometer data from the after-school segment is complex due to high day-to-day physical activity variability as children commute home from school using diverse travel modes and at variable times depending on their routines and after-school activity preferences.

The most mentally-loaded students in lessons do not compensate more for this load during recesses by PA than the less mentally loaded students. In developing habits of immediate compensation for mental load in lessons by PA during subsequent recesses, it is important to pay increased attention to boys and girls with higher mental load.

It is necessary to support the process of adopting habits by sufficient physical literacy of students, as well as teachers, and by changes in the school program.

## Figures and Tables

**Table 1:** The overall weekly physical activity and the average sedentary time on school and weekend days in participants according to the level of their school mental load.

Characteristics	VHML (*n* = 40)	HML (*n* = 34)	LML (*n* = 96)	VLML (*n* = 57)	H	*p*	*η^2^*
	Mdn/IQR	Mdn/IQR	Mdn/IQR	Mdn/IQR			
MET—min/week	3026/5755	4070/8283	4180/6665	3833/3045	4.90	0.179	0.022 *
Sitting—School Days	470/240	420/120	480/213	420/130	3.23	0.357	0.014 *
Sitting—Weekend	315/278	293/180	240/240	300/300	3.59	0.309	0.016 *

*Notes*: Mdn—median values; IQR—interquartile ranges; VHML—very high mental load; HML—high mental load; LML—low mental load; VLML—very low mental load; *ŋ^2^*—* 0.01 ≤ *ŋ^2^* < 0.06 small effect size.

**Table 2:** Physical activity intensity in participants with various levels of school mental load on weekends.

Characteristics of PA	VHML (*n* = 40)		HML (*n* = 34)		LML (*n* = 96)		VLML (*n* = 57)		H	*p*	*η^2^*
	Mdn	IQR	Mdn	IQR	Mdn	IQR	Mdn	IQR			
kcal·kg^−1^·h^−1^	1.49	1.15	2.32	1.72	2.07	2.05	2.01	1.84	7.22	0.065	0.032
steps·h^−1^ (number)	427	353	729	539	576	437	505	328	8.41 ^a^	0.038	0.037
physical inactivity (min·h^−1^)	40.42	10.43	35.77	10.97	38.03	10.27	39.17	11.61	4.84	0.184	0.021
≥3 METs (min·h^−1^)	1.21	1.84	3.48	3.62	2.13	2.71	2.08	3.13	9.66 ^a^	0.022	0.043
≥60 HRmax (min·h^−1^)	2.11	5.44	1.87	8.40	1.72	4.55	1.12	4.75	4.81	0.187	0.021

*Notes:* Mdn—median values; IQR—interquartile ranges; H—Kruskal-Wallis test; *ŋ^2^*—Cohen’s effect size; *p*—significance level; *ŋ^2^*—0.01 ≤ *ŋ^2^* < 0.06 small effect size; VHML—very high mental load; HML—high mental load; LML—low mental load; VLML—very low mental load; ^a^: significant difference between groups (VHML-HML).
